# The Determination of Importance of Sequences Neighboring the Psi Sequence in Lentiviral Vector Transduction and Packaging Efficiency

**DOI:** 10.1371/journal.pone.0050148

**Published:** 2012-11-21

**Authors:** Seon Hee Kim, Hyun Jeong Jun, Soo In Jang, Ji Chang You

**Affiliations:** 1 National Research Laboratory of Molecular Virology, Department of Pathology, School of Medicine, The Catholic University of Korea, Seoul, Republic of Korea; 2 Avixgen Inc., Seoul, Republic of Korea; Ghent University, Belgium

## Abstract

A number of lentiviral vector systems have been developed for gene delivery and therapy by eliminating and/or modifying viral genetic elements. However, all lentiviral vector systems derived from HIV-1 must have a viral packaging signal sequence, Psi (Ψ), which is placed downstream of 5′ long terminal repeat in a transgene plasmid to effectively package and deliver transgene mRNA. In this study, we examined feasible regions or sequences around Psi that could be manipulated to further modify the packaging sequence. Surprisingly, we found that the sequences immediately upstream of the Psi are highly refractory to any modification and resulted in transgene vectors with very poor gene transduction efficiency. Analysis around the Psi region revealed that there are a few sites that can be used for manipulation of the Psi sequence without disturbing the virus production as well as the efficiency of transgene RNA packaging and gene transduction. By exploiting this new vector system, we investigated the requirement of each of four individual stem-loops of the Psi sequence by deletion mapping analysis and found that all stem-loops, including the SL4 region, are needed for efficient transgene RNA packaging and gene delivery. These results suggest a possible frame of the lentiviral vector that might be useful for further modifying the region/sequence around the packaging sequence as well as directly on the Psi sequence without destroying transduction efficiency.

## Introduction

A number of lentiviral vector systems have been developed for gene delivery and therapy, as they have an advantage of delivering genes of interest efficiently into non-dividing cells compared to the conventional retroviral vector system [1], [2], [3]. Lentiviral vectors derived mostly from human immunodeficiency virus-1 (HIV-1) were engineered and modified extensively to either eliminate or substitute the genetic elements from the parental virus in order to avoid the possibility of generating replication-competent virus [4], [5], [6]. Naldini and colleagues generated a HIV-1-based lentiviral vector system in which the gag-pol genes were separated from the transgene plasmid carrying the gene of interest, and the HIV envelope protein was substituted with vesicular stomatitis virus (VSV) glycoprotein to widen the infection target range and give higher stability [7]. This first-generation lentiviral vector was then further modified by deleting four accessory genes, *vif*, *vpr*, *vpu*, and *nef*, as their contributions on virus replication were less crucial or cell type-specific, yielding a second generation of lentiviral vector [8], [9]. Further modification included replacing the Rev response element (RRE) component with the Mason-Pfizer monkey virus (MPMV) constitutive transport element (CTE) [9], [10], and removing the Tat gene by introducing a strong and constitutive cytomegalovirus (CMV) promoter in the transgene plasmid [9], [11]. The third generation of lentiviral vector system was also developed as a so-called self-inactivating viral vector, which delivers and expresses only the intended gene of interest, and reported to be unable to generate a full-length viral mRNA once integrated in a target cell chromosome due to the elimination of a region of transcriptional element U3 from the 3′-end of the long terminal repeat (LTR) [11], [12].

**Figure 1:**
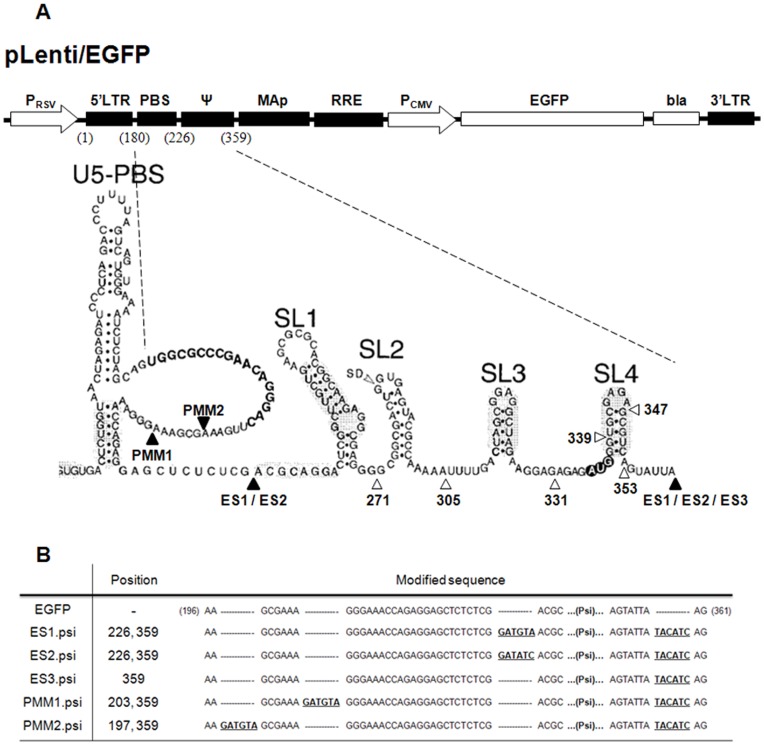
Schematic representation of lentiviral plasmids used in this study. A) The numbers in parentheses at the top designated as pLenti/EGFP indicate each region of viral sequence elements residing in the 5′ UTR of HIV-1 according to the genome sequence of HXB2 isolate [19]. Shown below is an enlargement of the 5′ UTR region and its nucleotide sequence. Black triangles around the packaging sequence Psi indicate nucleotide positions in which restriction enzyme sites were created to generate lentiviral transgene vectors used in this study. Open triangles indicate each nucleotide positions to generate serial deletion mutants of the Psi tested in this study, which are also schematically shown in Figure 6A. B) Nucleotide positions and sequence changes introduced in the transgene vector constructs are indicated.

However, none of the lentiviral vector systems developed to date have addressed any possible modifications on the so-called the viral packaging signal sequence, Psi (Ψ), which must be placed immediately downstream of the 5′ LTR in transgene plasmid in order to package and deliver a transgene mRNA. Psi (Ψ) is a highly structured RNA sequence, consisting of four stem-loop (SL) structures that has a strong affinity to the nucleocapsid (NC) domain of Gag and has been shown to be critical for viral genomic RNA packaging as well as gene delivery efficiency [13], [14], [15], [16], [17]. Here, we examined a possibility to modify the Psi sequence and whether any feasible sequences could be exploited for further modification of the packaging signal sequence. We found that the sequence immediately upstream of Psi is highly refractory to any changes, resulting in transgene vectors with very poor gene transduction efficiency. However, further analysis revealed that there are a few sites that can be used for further manipulation that did not disturb the virus production as well as gene transduction efficiency. Establishing this modified transgene vector allowed us to identify for the first time the contribution of each SL of the Psi in transgene mRNA packaging and transduction by a serial deletion analysis. It was found that the Psi sequence, including the SL4 region as a whole in the lentiviral vector is required for efficient transgene RNA packaging and gene delivery.

**Figure 2:**
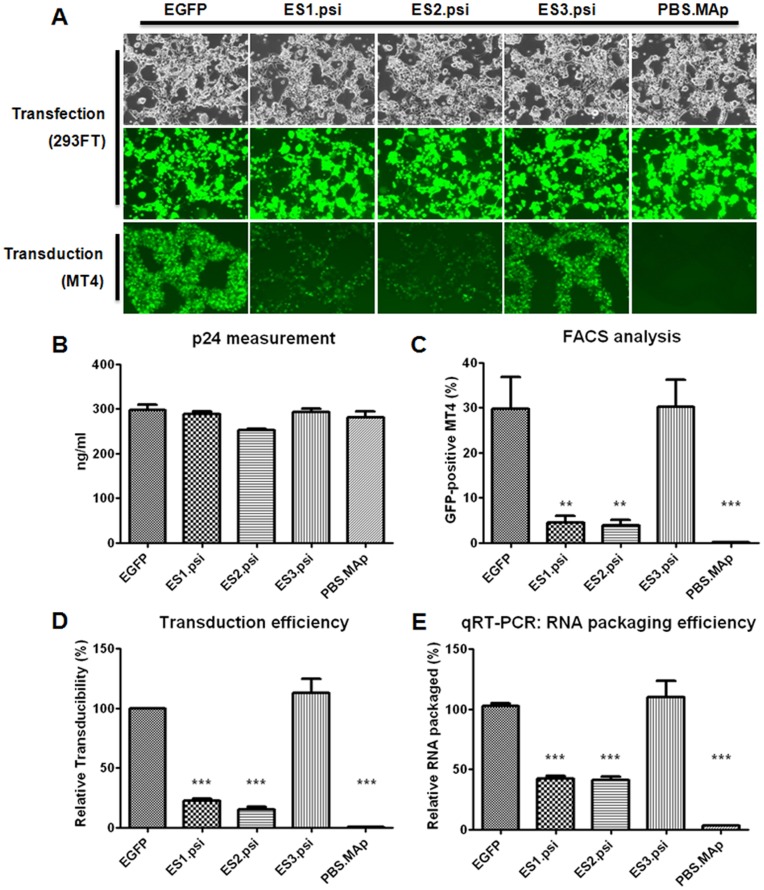
Transduction efficiency and RNA packaging of various lentiviral vectors. A) One milliliter of lentiviral supernatants harvested from transiently-transfected 293FT cells was used to transduce 1×10^5^ MT4 cells. Expression levels of the EGFP transgene were observed by fluorescence microscopy in 293FT cells and MT4 cells 30 hr post-transfection and 72 hr post-transduction, respectively. B) Amounts of virus particles in p24 in harvested viral supernatants were measured by ELISA. (error bars: ±S.E.M.) C) Percentage of EGFP positive MT4 cells after transduction was determined by FACS as described in Materials and Methods. D) The transducibility of each virus was calculated by normalization with p24 ELISA data, and each graph represents five different experiments performed independently. E) One microliter of viral supernatant was subjected to direct virus lysis and qRT-PCR to measure packaged viral RNA, and the results were normalized with the ELISA data. Statistically different results were tagged with two (p<0.05) or three (p<0.0001) asterisks (One-way ANOVA).

## Materials and Methods

### Plasmid Construction

pLenti/EGFP and pLenti/PBS.MAp transgene vectors were described previously [17] and used here as positive and negative controls, respectively. The following lentiviral transgene plasmids were newly designed and generated to characterize attribution of the Psi or other sequences. To generate pLenti/ES1.psi and pLenti/ES2.psi, the HIV-1 Psi sequence was amplified from pLenti/EGFP with the following two types of forward primers and one reverse primer (SnaBI_SL1_F: 5′-CCCTACGTAACGCAGGACTCGG-3′, EcoRV_SL1_F: 5′-CCCGATATCACGCAGGACTCGG-3′, SnaBI_SL4_R: 5′-GGGTACGTATAATACTGACGCTCT-3′), respectively, digested with its respective restriction enzymes, and inserted into pLenti/PBS.Map vector. As a result, pLenti/ES1.psi and pLenti/ES2.psi containing two different inserted enzyme sites at the 5′ end of the Psi sequence but with only three nucleotides differing between them were obtained (details in sequence difference are shown in Fig. 1B). The ES3.psi insert was obtained from pLenti/EGFP by PCR, using the same reverse primer used for pLenti/ES1.psi and a forward primer Lenti_PBS_F (5′-CGGCGCCCGAACAGGGACTT-3′). The amplified DNA fragments were treated with KasI and SnaBI. pLenti/PBS.MAp was used as backbone again, which was digested with the same enzymes, and then ligated with ES3.psi insert to generate pLenti/ES3.psi. To construct pLenti/PMM1.psi, 35-bp DNA fragments were amplified by PCR with Lenti_PBS_F and EcoRV_PMM_R (5′-CCCGATATCTTTCGCTTTCAA-3′), and inserted into pLenti/PBS.MAp after digestion with KasI and EcoRV restriction enzymes, resulting in a plasmid named as pLenti/PMM. This plasmid was treated with EcoRV and the Psi sequence amplified from pLenti/EGFP with SnaBI_PMM.psi_F (5′-GGGTACGTAGGGAAACCAGAG-3′) and SnaBI_SL4_R primers was inserted. pLenti/PMM2.psi was generated similarly, except the 29-bp DNA fragment insert used in the first step of cloning was synthesized from Genotech, (PMM2_F: 5′-GGGGGCGCCCGAACAGGGACTTGAAAGATATCGGG-3′, PMM2_R: 5′-CCCGATATCTTTCAAGTCCCTGTTCGGGCGCCCCC-3′) having KasI and EcoRV restriction enzyme sites at the ends of sequence and inserted into pLenti/PBS.MAp. Using the resulted pLenti/PMM2 as a backbone, the Psi sequence was amplified from pLenti/EGFP by PCR with SnaBI_PMM2.psi_F (5′-GGGTACGTAGCGAAAGGGAAACC-3′) and SnaBI_SL4_R inserted to generate pLenti/PMM2.psi. Alternative Psi sequences embedding SW8.4 or SW8.4.Mut in SL3 position of Psi were amplified from HIV-gpt-SELEX.SL3 or HIV-gpt-Mut-SELEX.SL3 (a gift from Dr. Parslow at Emory University, USA) by PCR and inserted into pLenti/PBS.MAp using the same cloning strategy of pLenti/ES1.psi to generate pLenti/ES1.SW8.4.psi and pLenti/ES1.Mut.psi. Likewise, the PCR products were also inserted into pLenti/ES3.psi, pLenti/PMM1, and pLenti/PMM2 to construct pLenti/ES3.SW8.4.psi and pLenti/ES3.Mut.psi, pLenti/PMM1.SW8.4.psi and pLenti/PMM1.Mut.psi, as well as pLenti/PMM2.SW8.4.psi and pLenti/PMM2.Mut.psi, respectively.

**Figure 3:**
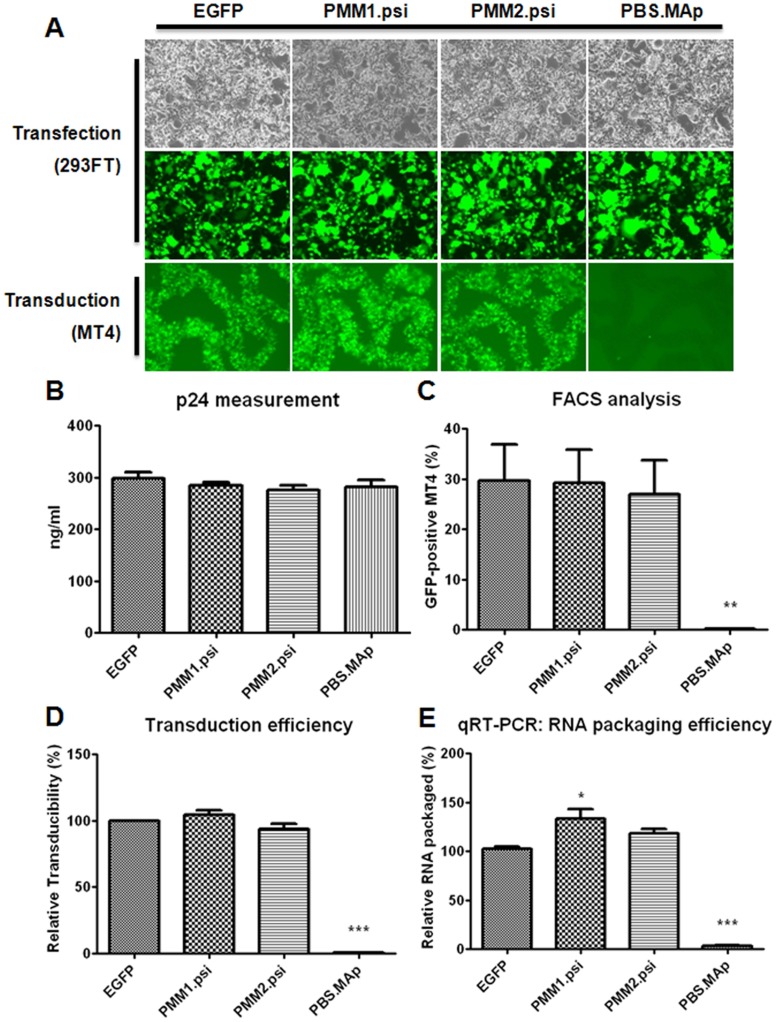
Transduction efficiency and RNA packaging of the PMM.psi transgene vectors. A) Lentiviruses harvested from transfected 293FT cells with pLenti/PMM1.psi or PMM2.psi were used for MT4 transduction, and the transduced cells were observed at 72 hr post-transduction. B) Amounts of virus particles in p24 in harvested viral supernatants were measured by ELISA, and C) Percentage of EGFP positive MT4 cells transduced was determined by FACS. D) Lentiviral transduction ratio was calculated from FACS and p24 ELISA results. Four independent experiments were performed. E) qRT-PCR was performed in triplicate as described in the legend of Figure 2, and the data were presented as p24 normalized ratios. All data were compared to Lenti/EGFP control in One-way ANOVA (F-test), and data showing significant differences (p<0.001) were marked with three asterisks.

**Figure 4:**
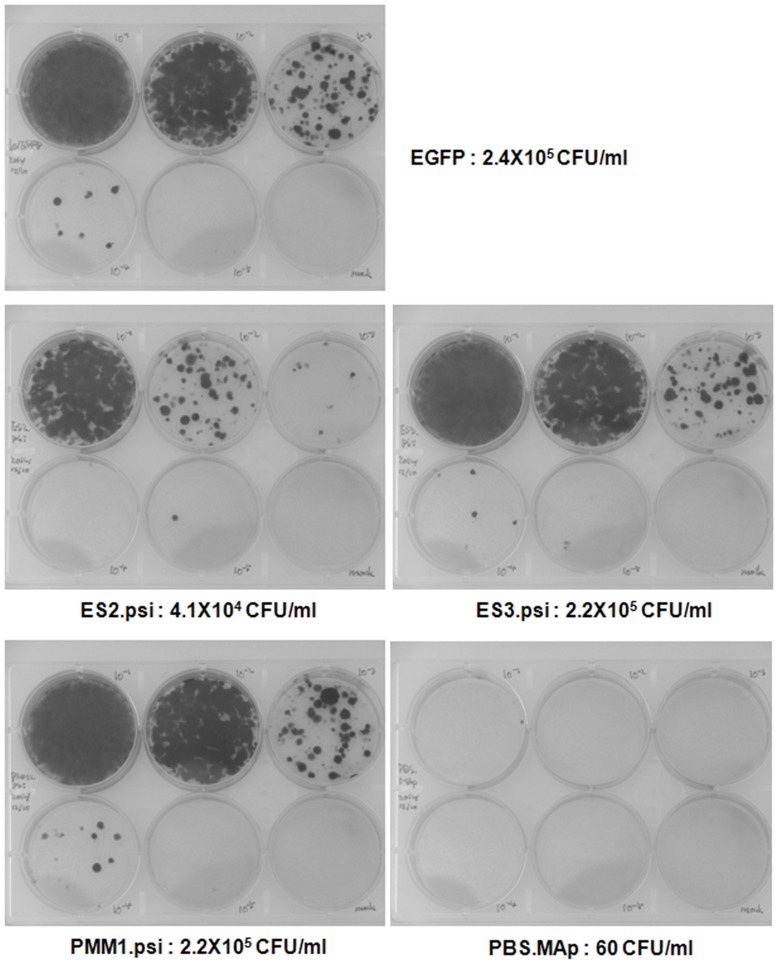
Titration of lentiviral titers using HT1080 cells. Harvested lentiviral supernatants were subjected to 10-fold serial dilution for 5 times, and then used to transduce HT1080 cells. These cells were cultivated under Blasticidin selection (5 µg/ml) for 14 days, and stained with crystal violet. The stained colonies in plates were counted and used to calculate the titers of lentiviral vectors, which were presented collectively in Table 1.

**Table 1:** Determination of lentiviral titers and transduction efficiency.

	p24 concentration(ng/ml)	HT1080 colony(CFU*/ml)	Transduction ratio(%)
EGFP	298±12	235,000	100
ES2.psi	252±3	41,333	18
ES3.psi	293±8	215,000	91
PMM1.psi	284±6	215,000	91
PBS.MAp	282±13	60	0.03

* CFU: Colony Forming Unit.

**Figure 5:**
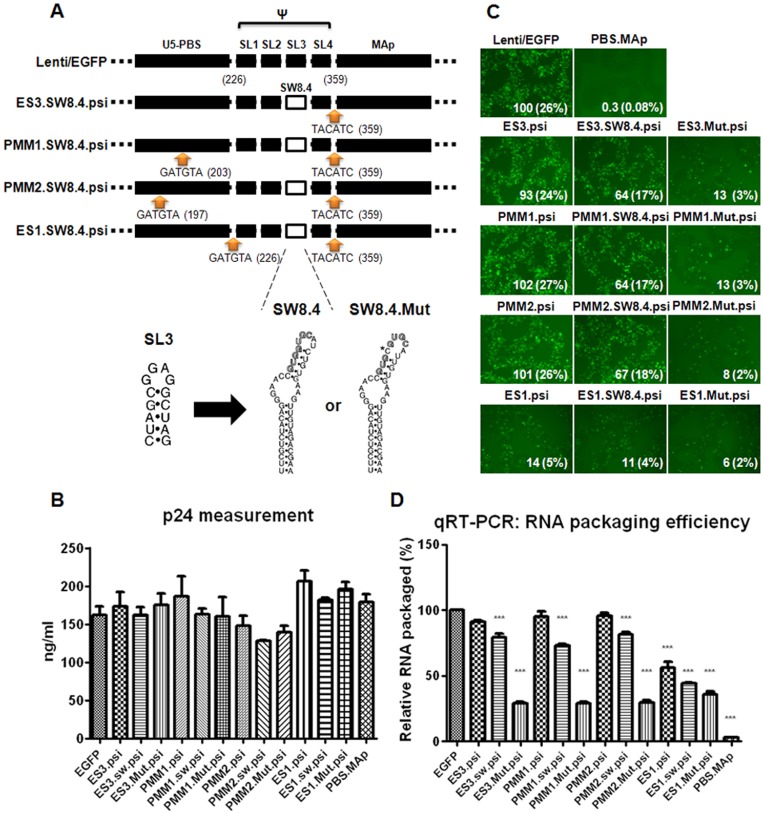
Transduction efficiency and RNA packaging by alternative Psi packaging sequence in various transgene vectors. A) Various transgene plasmids harboring SW8.4 or SW8.4.Mut sequence substituted in the SL3 position of HIV-1 Psi are schematically illustrated. B) Each mutant plasmid was transfected into 293FT cells with packaging plasmids, and the harvested supernatants were determined for viral p24 values and used to transduce MT4 cells. C) Percentage of EGFP-positive MT4 cells from FACS analysis are shown in parenthesis in the figures and the numbers in each figure indicate the relative transducibility of each vector compared to that of pLenti/EGFP transgene vector. D) The viral RNA packaging efficiency was measured by qRT-PCR and normalized to p24 ELISA data. All data are presented in comparison to the EGFP control in One-way ANOVA, and data showing significant differences (p<0.001) are marked with three asterisks.

**Figure 6:**
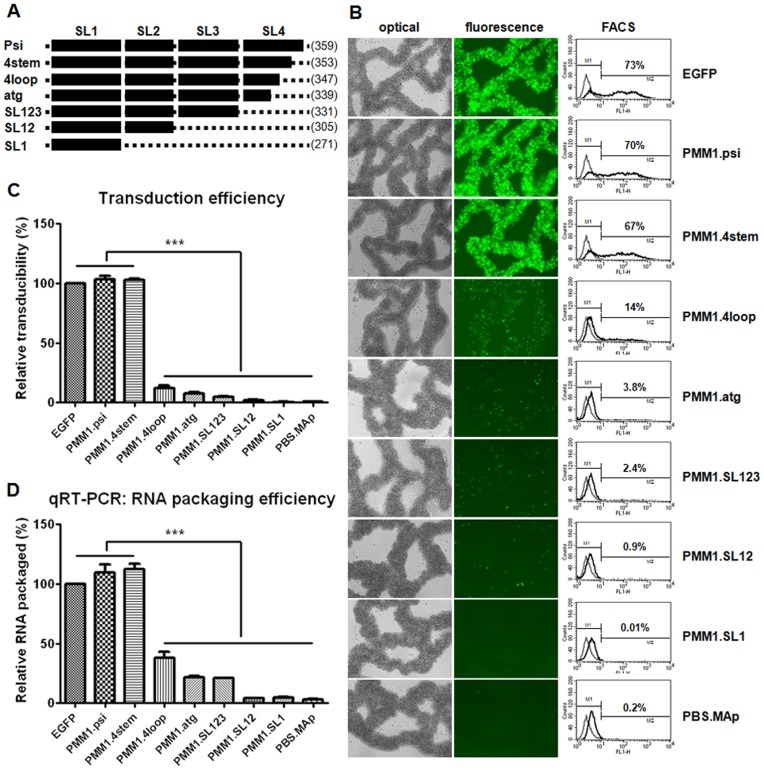
The effect of serial deletions of the Psi sequence on gene transduction efficiency and RNA packaging. A) Psi sequence of PMM.psi vector was deleted from 3′ end serially. The nucleotide position numbers were also shown in parentheses at the end of each mutant constructs. B) Lentiviruses produced from PMM1.psi deletion mutants were transduced to MT4 cells for 72 hr, and expression of EGFP was visualized by fluorescence microscopy and percentage of EGFP positive cells was quantified with FACS and indicated on each histogram. C) Transduction efficiency of each vector in MT4 cells was calculated from FACS data normalized with p24 ELISA. D) Viral RNA packaging efficiency was determined from qRT-PCR and p24 ELISA. All data are presented in comparison to EGFP control in One-way ANOVA, and data showing significant differences (p<0.001) are marked with three asterisks.

To generate transgene vectors with serial deletion mutants from the 3′ end of the Psi, two PCR steps were employed with six reverse primers (ppD_4stem_R: 5′-CTAATTCTCCCCCGCTTGACGCTCTCGCAC-3′, ppD_4loop_R: 5′-CTACTTCTCCCCCGCTTCTCGCACCCATCT, ppD_atg_R: 5′-CTAATTCTCCCCCGCTCCCATCTCTCTCCT-3′, ppD_SL3_R: 5′-CTAATTCTCCCCCGCTCTCCTTCTAGCCTC-3′, ppD_SL2_R: 5′-CTAATTCTCCCCCGCTTTTTTGGCGTACTC-3′, ppD_SL1_R: 5′-CTAATTCTCCCCCGCTCCCTCGCCTCTTGC-3′), SnaBI_PMM.psi_F as forward primer, and one common reverse primer (ppD_EcoRV_R: 5′-GGGGATATCCGCGATCTAATTCTCC-3′). The first step of PCR was performed with SnaBI_PMM.psi_F and one of six reverse primers for 25 cycles, and 5 µl of first step PCR was subjected to a second PCR step. In the second PCR step, SnaBI_PMM.psi_F and ppD_EcoRV_R were used as a primer set for another 25 cycles, and the resulting six different inserts amplified were treated with SnaBI and EcoRV and ligated into pLenti/PMM after digestion with EcoRV. The resulting plasmids were named pLenti/PMM1.4stem, pLenti/PMM1.4loop, pLenti/PMM1.atg, pLenti/PMM1.SL123, pLenti/PMM1.SL12, and pLenti/PMM1.SL1. All the engineered constructs were verified by DNA sequencing. These lentiviral transgene plasmids carry in common an EGFP transgene and a blasticidin selection marker gene as described [17].

### Cell Culture and Transfection

293FT cells (Invitrogen) were maintained in 10% fetal bovine serum, 1% penicillin/streptomycin, and 1% non-essential amino acids in Dulbecco’s modified Eagle medium (DMEM) at 37°C and 5% CO_2_. Twenty-four hours before transfection, 1×10^6^ 293FT cells were seeded into 6-well plates. The following day, 3 µg of a mixture of pLP1, pLP2, pLP/VSVG plasmids encoding viral proteins Gag-Pol, Rev, and VSV-G (Invitrogen) and 1 µg of lentiviral transgene plasmids were transfected into each well for lentivirus production using Lipofectamine 2000 (Invitrogen). Five to six hours after transfection, the DNA-reagent mixture was removed from the plates and 2 ml fresh DMEM with 100 mg/L sodium pyruvate was added to wells. At 30 hrs posttransfection, lentiviral supernatants were harvested and filtrated with 0.45-µm filters. MT4 cells were maintained in RPMI 1640 media with 10% fetal bovine serum and 1% penicillin/streptomycin at 37°C and 5% CO_2_. HT1080 cells were maintained in DMEM supplemented with 10% fetal bovine serum, 1% penicillin/streptomycin, and 1% non-essential amino acids at 37°C and 5% CO_2._

### Lentiviral Transduction

To determine transduction efficiency, MT4 cells were used to transduce with lentivirus harvested from 293FT cell transfection. MT4 cells (10^5^) were counted, placed in wells of 48-well plates, and inoculated with 1 ml viral supernatants above. The transduced cells were incubated at 37°C for 2–3 hrs, and then the supernatants were removed and replaced with same volume of fresh RPMI. At 72 hrs post-transduction, GFP-positive MT4 cells were observed by fluorescence microscope qualitatively and quantitatively analyzed by fluorescence-activated cell sorting (FACS).

### Determining Virus Amount by p24 ELISA

Ten microliters of each harvested viral supernatant were diluted 100-fold twice with phosphate buffer. To measure p24 concentration of the viral supernatants, 100 µl of the diluted virus were added to wells of an enzyme-linked immunosorbent assay (ELISA) kit (Advanced Bioscience Laboratories) in duplicate. After one hour incubation at 37°C, each well was washed with 200 µl phosphate buffer four times, then 100 µl horseradish peroxidase-conjugated anti-HIV-1 p24 antibody were added and incubated at 37°C for another 1 hr. Wells were washed again and incubated at room temperature for 30 min with 100 µl substrate (tetramethylbenzidine). One hundred microliters of 2N sulfuric acid were added to stop the reaction, and absorbance was measured at 450 nm. Positive and negative controls were used to quantify absolute amount of p24 antigen of each virus according to the manufacturer’s instructions.

### FACS Analysis

Transduced MT4 cells were analyzed using a FACSort instrument (Becton Dickinson) to determine the green fluorescence protein (GFP)-positive cell ratio. Each sample was centrifuged at 7000 rpm for 5 min, resuspended in 1 ml 4% paraformaldehyde solution, and subjected to FACS.

### qRT-PCR

To detect encapsidated transgene mRNA in virions, viral supernatants were directly subjected to a qRT-PCR reaction without further manipulation to avoid any experimental errors that might be caused by RNA preparation or quantification. First, 40 µl viral supernatants were treated with DNase I (Promega) and incubated at 37°C for 1 hr, and then the reaction was ended with 5 µl EDTA stop solution. One microliter of the DNase I-treated viral supernatants was added to HotStart-IT SYBR Green One-Step qRT-PCR master mix (USB), with 2 mM DTT and 0.4% Triton-X 100 to disrupt virus in the qPCR mix. Forty-five cycles of qPCR were performed with a Roche LightCycler 480 with the following primers: EGFP-QPCR-F: 5′-ACGACGGCAACTACAAGACC-3′ and EGFP-QPCR-R: 5′-TGTAGTTGTACTCCAGCTTGTGC-3′. Results were analyzed with LC480 software and normalized by p24 amounts determined by ELISA when necessary.

### Lentiviral Titer Determination

One hundred thousand HT1080 cells were placed in 6-well plates and incubated at 37°C in a 5% CO_2_ incubator overnight. On the following day, lentiviral supernatants were serially diluted in 10-fold for five times (from one-tenth to 10^−5^) in a total volume of 1 ml and added to wells with a 6 µg/ml final concentration of Polybrene (Sigma). After incubation at 37°C overnight, the medias containing diluted viral supernatants were replaced with 2 ml of DMEM. The next day, medias were changed again to fresh DMEM containing blasticidin (5 µg/ml final concentration; Sigma) and the cells were incubated at 37°C for 14 days with the same media changes in every other day. After 14 days of selection, each well was washed twice with 1 ml PBS and incubated with 1 ml of crystal violet solution [1% crystal violet (Sigma) and 10% ethanol] for 10 min at room temperature. Then the cells were washed with 1 ml PBS four times to remove excessive crystal violet, and stained colonies were counted for titration.

## Results

To construct a new transgene vector that harbors restriction enzyme sites around the Psi packaging sequence yet retains gene transduction efficiency, we generated the lentiviral plasmids pLenti/ES1.psi, pLenti/ES2.psi, and pLenti/ES3.psi as depicted in Figure 1. pLenti/ES1.psi contains SnaBI sites at both ends of the Psi sequence, whereas pLenti/ES2.psi contains EcoRV and SnaBI at the 5′ and 3′ ends of the Psi sequence, respectively. For comparison, another transgene vector pLenti/ES3.psi was also constructed, which contained only one SnaBI enzyme site inserted at 3′ end of the Psi just as in the pLenti/ES1.psi and pLenti/ES2.psi, but the 5′ end of the Psi was kept intact. The individual plasmids were transiently transfected into 293FT cells with helper plasmids and the resulting viruses were harvested after 30 hr. pLenti/EGFP with intact Psi sequence and pLenti/PBS.Map, which does not have a Psi sequence as described previously [17], were also subjected to the same analysis as controls. For each of the transfection reactions, similar transfection efficiencies as well as virus particle production were observed (Fig. 2A upper panel and B), indicating that the expression of transgene EGFP and viral protein expression were not affected by mutations of the engineered lentiviral transgene vectors. Equal volumes of the harvested viruses were used to transduce MT4 cells. At 72 hr after transduction, the number of EGFP-positive MT4 cells were examined by fluorescence microscopy and counted quantitatively by FACS (Fig. 2A lower panel and C), and the FACS signals were normalized with viral p24 data determined by ELISA to obtain the relative transduction efficiency of each of the lentiviral transgene vectors constructed (Fig. 2D). Surprisingly, rather dramatically differing results were observed among the transgene plasmid constructs. ES1.psi and ES2.psi transgene vectors showed much lower transduction efficiency, at approximately 20% of the EGFP vector control, whereas the ES3.psi transgene vector showed almost equal transduction efficiency compared to the EGFP vector control. PBS.MAp, a negative control vector for the dependency of the Psi sequence, showed no notable EGFP gene transduction. To further determine the packaging efficiency of the transgene mRNA *per se*, we directly measured the amount of EGFP transgene mRNA from 1 µl of each viral supernatant using quantitative RT-PCR, and the data obtained from qRT-PCR were normalized by their p24 values determined by ELISA assay. Similar to the patterns of transduction efficiency in Fig. 2D, a very low level of transgene mRNA packaging was observed in the PBS.MAp vector, compared to the positive control Lenti/EGFP vector. Viruses obtained from both ES1.psi and ES2.psi transfection also have lower ratios of transgene mRNA packaging compared to the control. In contrast, ES3.psi derived virus shows a ratio as high as the positive control (Fig. 2E), which was consistent with the transduction efficiency data (Fig. 2D). Together, these results indicate that transgene transduction efficiency directly depends on the efficiency of RNA encapsidation and suggest that modification in the sequence immediately upstream of the Psi sequence changes the efficiency of RNA packaging, which in turn utterly affects the gene transduction efficiency of the lentiviral vector. On the other hand, changes at the 3′ end of Psi are relatively tolerable.

In light of previous studies suggesting that the sequences in front of the Psi may form weak stems either with U5 region or be flanked between stems in whole HIV-1 genome [18], [19], [20], [21], [22], the changes we have made at the 5′ end of the Psi region might disrupt RNA structures located immediate upstream of Psi and thus affecting RNA encapsidation efficiency. Thus, we reasoned that introducing restriction enzyme sites into a less structured region of 5′ LTR might help not to affect the efficiency of RNA packaging and maintain efficient gene transduction. To this end, we engineered a restriction enzyme site in a position further upstream of the Psi sequence, which is in a loop structure located immediately downstream of Primer Binding Site (PBS) as shown in Fig. 1. The first vector, PMM1.psi, has two restriction enzyme sites inserted in a sequence position 203 as for the 5′ site and in the same 3′ site position as used for the ES series above. The transducibility and RNA packaging efficiency of PMM1.psi transgene vector were measured. Different from ES1.psi or ES2.psi, placing an enzyme site into the PBS loop did not affect EGFP gene transduction efficiency as shown in the lentivirus-transduced MT4 cell FACS analysis (Fig. 3A–D) and RNA packaging efficiency determined by RT-qPCR analysis (Fig. 3E). The PMM1.psi vector showed even a slightly higher viral RNA packaging efficiency than that of Lenti/EGFP control vector, indicating a loop structure of PBS region is tolerable for sequence modification, unlike the ES1.psi or ES2.psi vectors. We then generated another insertion mutation vector named PMM2.psi that has a restriction enzyme site further upstream in position 197 (Fig. 1), in order to confirm further that the loop region is really tolerable to sequence changes. Again, PMM2.psi vector also did not show any difference in transgene transducibility as well as RNA packaging efficiency from that of the Lenti/EGFP control and of PMM1.psi (Fig. 3). These results were further confirmed by a viral titration assay using HT1080 cells as shown in Figure 4 and Table 1, where it was demonstrated again that while the ES3.psi or PMM1.psi vectors have a similar transduction efficiency to Lenti/EGFP control, the ES2.psi vector show significantly lower transduction efficiency than the control.

Having identified a lentiviral transgene vector that is now possible to manipulate sequences around the Psi packaging signal without affecting transgene transducibility, we next wanted to see if the gene transduction efficiency of these PMM1.psi or PMM2.psi vectors could be exploited further by introducing a sequence other than Psi into the vectors. We employed an alternative packaging sequence, known as SW8.4, which was identified previously as a high-affinity RNA aptamer to HIV-1 NC and showed to be functional in viral genomic RNA packaging as a Psi substitute [23]. The alternative packaging signal SW8.4 sequence as well as its mutant type SW8.4.Mut embedded in SL3 position of the Psi were introduced into all our lentiviral transgene plasmids respectively, resulting in plasmids ES1.SW8.4.psi and ES1.Mut.psi (all derived from ES1.psi), ES3.SW8.4.psi and ES3.Mut.psi (from ES3.psi), PMM1.SW8.4.psi and PMM1.Mut.psi (from PMM1.psi), and PMM2.SW8.4.psi and PMM2.Mut.psi (from PMM2.psi). The organization of these lentiviral plasmids is schematically depicted in Figure 5A. Each of these individual transgene plasmids was tested for gene transduction efficiency. The transducibility of four different types of vectors harboring SW8.4.psi in the transgene constructs mostly followed the transducibility as well as packaging efficiency patterns of their parental vectors (Fig. 5B, C and D). Compared to Lenti/EGFP control, ES1.SW8.4.psi vector showed a low transduction rate of approximately 11%, as was its parental ES1.psi vector showed 14% of the control transduction rate. On the other hand, ES3.SW8.4.psi, PMM1.SW8.4.psi, and PMM2.SW8.4.psi all showed a similar transducibility of more than 60% of the Lenti/EGFP control and that of their parental vectors. The specificity of gene transduction efficiency of the vectors containing SW8.4.psi was verified by the observations that all of the SW8.4.Mut.psi mutants have transduction efficiency approximately 10% of Lenti/EGFP control, regardless of the difference in transducibility of their parental vectors. Together, these results indicate that PMM1.psi or PMM2.psi vectors are able to maintain their transducibility quite effectively even when additional changes are made on the packaging signal sequence.

Previously, we reported that the Psi sequence is absolutely required for efficient transgene packaging and transduction [17]. Yet, the exact level of contribution of each of the stem-loops in the Psi remains an important question. To address this issue, we exploited the PMM vectors further to determine the relative importance of each stem-loop. Deletion mutants were generated by serial deletion of 3′ end of the Psi, as shown in Figure 6A and open triangles in Figure 1 for their positions. pLenti/PMM1.4stem has six nucleotides deleted from the end of the Psi, and pLenti/PMM1.4loop has a deletion of the 3′ stem sequence to the last nucleotide of Psi. Each stem-loop was then removed one by one to generate PMM1.SL123, PMM1.SL12, and PMM1.SL1. For pLenti/PMM1.atg, all Psi sequence right after the Gag start codon was deleted. When we tested the packaging and transduction efficiency of these mutant vectors, we found a sharp reduction when 3′ part of SL4 stem was removed from the Psi as in the case of PMM1.4loop vector. Thereafter, transduction efficiency declined a little further with each deletion (Fig. 6B and C). Reduced gene transduction efficiency was well correlated to decreases in viral RNA encapsidation, as presented in Figure 6D. Viral RNA of the mutant vectors was less packaged according to the shortened Psi sequence length. This significant reduction in transduction caused by the SL4 stem deletion is rather unexpected, because SL4 has been suggested previously to have only weak affinity to NC [24], [25] and the deleted part of SL4 in PMM1.4loop was barely involved in any form of long-range interaction [20], [21], [26], [27]. However, our results strongly suggest that sustaining intact stem-loop structures of Psi including SL4 appears to be indispensible for proper and efficient transgene RNA packaging.

## Discussion

In this study, we investigated a lentiviral gene transduction vector system with a modified and alternative packaging signal Psi. The biosafety issues associated with using lentiviral transgene transduction vectors might depend on how the native viral sequences and genetic elements can be eliminated or substituted while preserving transduction ability. In addition, one advantage of using lentiviral vector system for studying of packaging mechanisms and processes is that influence from anything other than the HIV-1 UTR sequence harboring the packaging signal would be minimized in the encapsidation process. Thus, the results reflect a simple but more precise role of the packaging signal in gene transduction and the effects of any modification of the Psi signal sequence. We found that modifying the region immediately upstream of Psi resulted in highly diminished gene transduction efficiency. On the other hand, sequence modification in the far upstream region of Psi such as the PBS loop did not affect gene transduction efficiency as observed in PMM1.psi or its derivatives. These results concur to some extent with previous studies where mutations in the upper stem or loop of U5 region close to the primer binding site showed little effect on the RNA packaging efficiency, whereas changes to the lower stems close to Psi disrupted RNA packaging [19], [28], [29]. PMM vectors generated in this study showed tolerance for additional modifications, such as PMM1.SW8.4.psi vector that showed no differences in transducibility from that of ES3.SW8.4.psi. The transducibility of this SW8.4-harboring transgene vector was 65–70% of that of their parental vectors containing only wild-type Psi, which was a little lower than in previous study [23]. However, considering full-length genomic RNA was used to examine the packaging efficiency of SW8.4 in the above reference, the lower transducibility might be due to any excluded viral components or factors.

The SL4 deletion mutation in Psi illustrates the importance of preserving intact stem-loop structure for efficient viral genomic RNA packaging and transduction, although the mechanism for the involvement of SL4 stem in RNA packaging remains to be further understood. Likewise, we have also generated another series of deletion mutants in which the deletion was started from 5′ end of the Psi. The deletion of SL1 resulted in significantly lower transduction efficiency, below 10% of control. Further deletions showed worse transduction efficiency (data not shown), confirming that the intact stem-loop structure of the Psi is very important for gene transduction efficiency of lentiviral vector.

In summary, our results show for the first time that it is possible to modify lentiviral vectors using the region/sequence identified upstream of the Psi packaging sequence as well as directly on the Psi in the modified context without diminishing transduction efficiency. This finding brings us a step closer toward further generating a manipulable lentiviral vector system and could serve as a framing lentiviral vector for such development.
